# Accuracy of individual mandibular motion records using intraoral scanner for fixed implant- supported prosthesis designs: a comparative study

**DOI:** 10.1186/s12903-025-06282-x

**Published:** 2025-06-02

**Authors:** Bundhit Jirajariyavej, Panchanit Ounvorawong, Suchaya Pornprasertsuk-Damrongsri, Porntida Visuttiwattanakorn, Salisa Sriyarun, Pobploy Petchmedyai

**Affiliations:** 1 Department of Prosthodontics, Faculty of Dentistry, Mahidol University, Bangkok, Thailand; 2 Dental Implant Center, Faculty of Dentistry, Mahidol University, Bangkok, Thailand; 3 Department of Oral and Maxillofacial Radiology, Faculty of Dentistry, Mahidol University, Bangkok, Thailand

**Keywords:** Patient-specific motion, Individual mandibular motion, Occlusal registration, Dynamic occlusion, Eccentric movement

## Abstract

**Background:**

Accurate occlusal registration is critical for successful dental implant treatments. Traditional mechanical articulation provides insight into occlusal relationships but cannot replicate complex mandibular movements accurately. CAD/CAM advancements, including virtual articulators, enable dynamic occlusion analysis using either average-value settings or individualized mandibular movement data, depending on the system used. Patient Specific Motion (PSM) technology, using intraoral scanners to record actual mandibular movements, offers enhanced functional occlusal design. Despite these advancements, challenges such as patient movement and limited residual dentition affect accuracy, highlighting the need for further research into dynamic occlusal registration methods. This study investigates the impact of different occlusal registration techniques on prosthesis design under varying conditions of missing teeth.

**Methods:**

Ten participants were examined in an *in-vivo* study comparing the accuracy of three occlusal registration methods: 1) Static Occlusal Registration (SA), Dynamic Occlusal Registration including 2) Virtual Articulator (VA), and 3) Patient-Specific Motion (PSM). The tested models were simulated by digital tooth removal in three conditions of missing teeth, and 3-D deviations of occlusal contacts recorded before (reference) and after (tested) virtually tooth removal were assessed to reflect the accuracy of occlusal morphology reproduction.

**Results:**

The root mean square (RMS), and percentage of In-Tolerance and Out-of-Tolerance of divided occlusal surface areas were analyzed and visualized using a color-coded deviation map. Statistically significant differences were observed between static and dynamic methods (*p* < 0.05), though no significant differences between the two dynamic methods. The PSM method exhibited the highest RMS values and a trend of negative deviations, while the VA method demonstrated the largest positive deviations. In terms of the effect of missing teeth, greater deviations were observed in models with more extensive tooth loss, particularly at the functional cusp areas, suggesting reduced accuracy in complex occlusal conditions.

**Conclusions:**

This study confirms that different occlusal registration methods (STA, VA, PSM) significantly influence prosthesis design, with all showing clinically acceptable outcomes. PSM demonstrated advantages in customization and may improve treatment precision. Occlusal accuracy declined as the number of remaining teeth decreased, highlighting the importance of maintaining sufficient tooth contacts for reliable digital prosthesis design in partially edentulous cases.

**Trial registration:**

This study was retrospectively registered and obtained approval from the TCTR Committee (TCTR20241116001, Date: 16/11/2024).

**Supplementary Information:**

The online version contains supplementary material available at 10.1186/s12903-025-06282-x.

## Introduction

The success of dental implant treatments is closely associated with the proper management of occlusal forces. The primary goal of implant occlusion is to replicate a natural, physiological occlusion that promotes harmonious interactions between the upper and lower teeth, thereby avoiding excessive forces on the implant and surrounding tissues. This consideration is essential to preventing implant-related complications, including fractures of the implant prosthesis, loosening of the abutment screw, and implant failure. Unlike natural teeth, dental implants lack periodontal ligament support, making them unable to adapt to occlusal force changes. Furthermore, the materials commonly used in implant-supported restorations, which differ in mechanical properties from natural teeth, necessitate a more precise occlusion to ensure a long-term stability and functionality of the implant.

Conventional techniques, such as using a mechanical articulator with bite registration and facebow transfer, allow clinicians to evaluate both static and dynamic relationships between the upper and lower jaws. These techniques offer valuable insights for creating comprehensive treatment plans by helping to identify occlusal issues not visible intraorally [1]. However, mechanical articulators cannot fully replicate the complexities of biological movements, such as mandibular elasticity and muscle-guided chewing patterns, leading to limitations in accurately reproducing functional occlusion [2].

The integration of computer-aided design and manufacturing (CAD/CAM) technologies has improved occlusal registration processes, allowing for more precise and practical virtual articulation than mechanical articulators [3, 4]. Virtual articulators in CAD software enhance occlusal design by simulating mandibular movements, either through average-value settings or individualized motion data, depending on the system and integration. However, when relying on average settings, limitations in replicating functional mandibular movements accurately may occur. Deviations in virtual articulation may impact occlusal accuracy, necessitating additional chairside adjustments [5], and emphasizing the need for more advanced, individualized methods to improve functional outcomes.

In recent years, new techniques have been developed to incorporate personalized functional movements into the design of occlusal morphology by tracking mandibular motion using optical devices. One such system is the Patient Specific Motion (PSM) (3Shape A/S, Copenhagen, Denmark) which allows for personalized occlusal design by tracking mandibular movements through intraoral scanner. During eccentric movement, the PSM feature records the patient’s dynamic occlusion. The recorded digital data is employed to analyze mandibular movements and to adjust any possible occlusal errors in the occlusal design through CAD software. Consequently, the PSM feature helps minimize occlusal interference during eccentric movements [6]. However, the accuracy of the PSM feature may be affected by factors such as patient movement, scanner calibration, and operator skill, with additional costs and training required.

Despite the growing interest in incorporating patient-specific functional movements into the occlusal design, there is a notable scarcity of studies investigating the clinical outcomes and accuracy of newer technologies like the PSM feature. One of the few studies addressing dynamic occlusion in virtual articulation is by Li et al., 2021 [7], who explored the effects of different residual dentitions on the dynamic registration of wear facet morphology for a single mandibular first molar crown using a virtual articulator. Their findings highlight the limitations of average-value articulation in cases with insufficient residual reference teeth. While this study provides valuable insights, it underscores the need for more comprehensive approaches that incorporate individual mandibular movement data to improve occlusal design accuracy. Limited data exists regarding its reliability in routine practice or its impact on long-term prosthesis functionality. This gap in the literature highlights the need for further research to validate the clinical utility of PSM and virtual articulators.

Building on the gaps identified in previous research, this study investigates the impact of various occlusal registration methods on prosthesis design in simulated conditions of missing teeth, with a null hypothesis that there were no significant differences in prosthesis design among different occlusal registration methods and conditions of missing teeth.

## Methods

### Ethical regulation

The present investigation was carried out after receiving approval from the Research Ethics Committee of the Faculty of Dentistry, Mahidol University where the study was performed (COA.No.MU-DT/PY-IRB 2023/052.0109, Date of approval 01/09/2023).

### Sample size calculation

The sample size was calculated using the PASS power program (Power Analysis and Sample Size, 2021, v21.0.3, NCSS, Kaysville, Utah, United States) based on root mean square and standard deviations from the previous study of Li et al., 2023 [8], based on the significance level of 5% and 80% study power. The total sample size was 10 subjects.

### Criteria for sample selection

A total of 10 participants, all over 18 years of age with fully erupted permanent dentition and intact occlusal surfaces, were included in the study. Participants had no active carious lesions or other dental defects and displayed no signs of malocclusion or temporomandibular disorders. Each participant demonstrated group function occlusion on at least one side. Participants were recruited from the Faculty of Dentistry, Mahidol University, Thailand. Exclusion criteria included parafunctional habits, the presence of third molars or partially erupted teeth that interfered with normal occlusion, and unwillingness to participate in the study.

### Procedures

#### Oral examination

Participants underwent an intraoral examination to determine if they met the inclusion criteria. The examination ensured at least three occlusal contact points distributed bilaterally on the posterior teeth in maximum intercuspation (MIP) without interferences during function.

#### Digital impression

The Intraoral scanner (TRIOS 3; 3Shape A/S, Copenhagen, Denmark) was used to acquire digital impressions of the mandibular and maxillary teeth, along with buccal scans and the patient’s jaw motion. To record PSM at the site that has a group function occlusion, the protrusive and laterotrusive movements were captured by positioning the wand tip on the buccal surfaces of the first molars and adjacent teeth. All scan data were exported in the standard tessellation language (STL) format, and the orders were saved in the 3Shape Order Exchange (3OXZ) file format as the original records. The original occlusal contacts of each participant, obtained before any virtually tooth removal, served as the control group or reference for subsequent 3D deviation analyses.

#### Manipulation of digital dentition and prosthesis design

The 3Shape Dental System (3Shape A/S, Copenhagen, Denmark), including the Model Builder and Implant Studio modules was used to manipulate digital dentition by virtually removing teeth to create edentulous spaces, simulating clinical scenarios of partial edentulous. Virtual implant analogs were then placed within these spaces to facilitate prosthetic design.

Three models simulating different conditions of missing teeth were generated by virtually removing selected teeth:

- Model 1: tooth 36 or 46
- Model 2: tooth 35–37 or 45–47
- Model 3: tooth 35–37 and 45–47

Prosthesis models were designed in CAD software program (3Shape Dental System v2019; 3Shape A/S, Copenhagen, Denmark), the scanned files in 3OXZ format were imported and categorized as implant-supported crowns. Occlusal morphology and crown contour were adjusted to align anatomically with adjacent and opposing teeth. Occlusal contact points were identified using the color-coded contact map within the CAD system. The identified occlusal contacts were then elevated by 0.3 mm using “Sculpting tool” in CAD software, as described in a previous study [7]. This procedure was performed consistently by a single trained operator. The final crown designs, with elevated contact locations, were exported in STL format for further analysis.

#### Occlusal reproduction

**Fig. 1:**
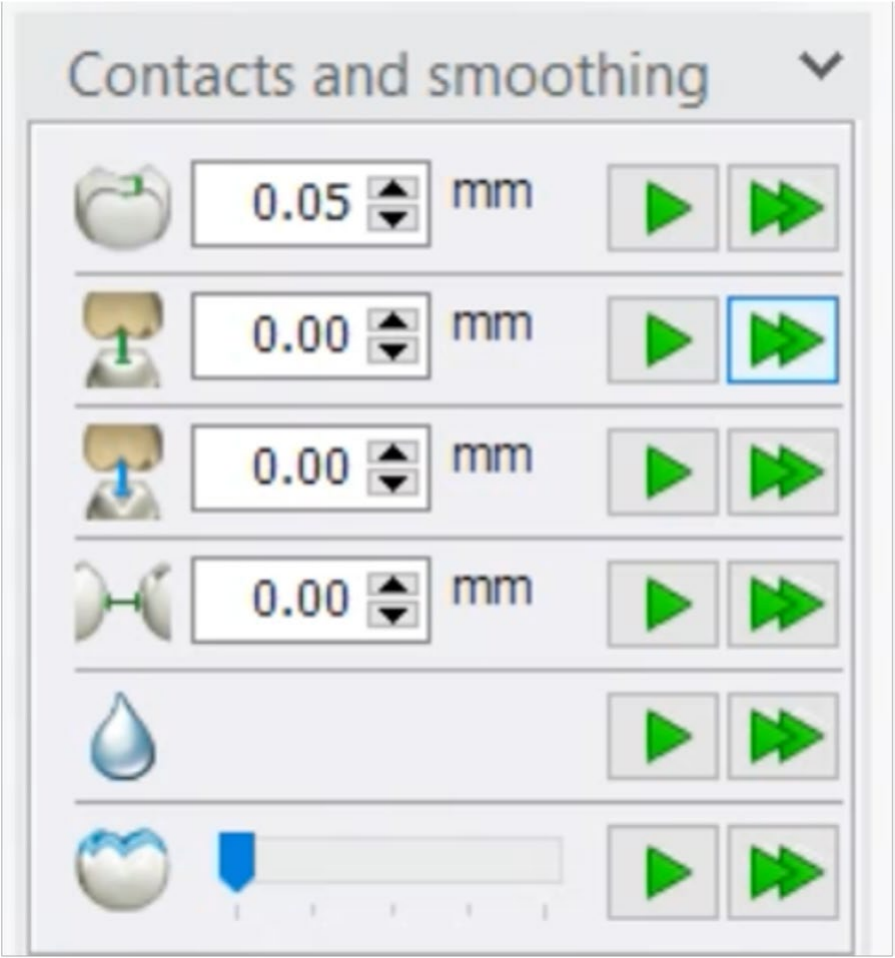
Screenshot from CAD software displaying the “Contacts and Smoothing” tool used for static occlusal registration

**Fig. 2:**
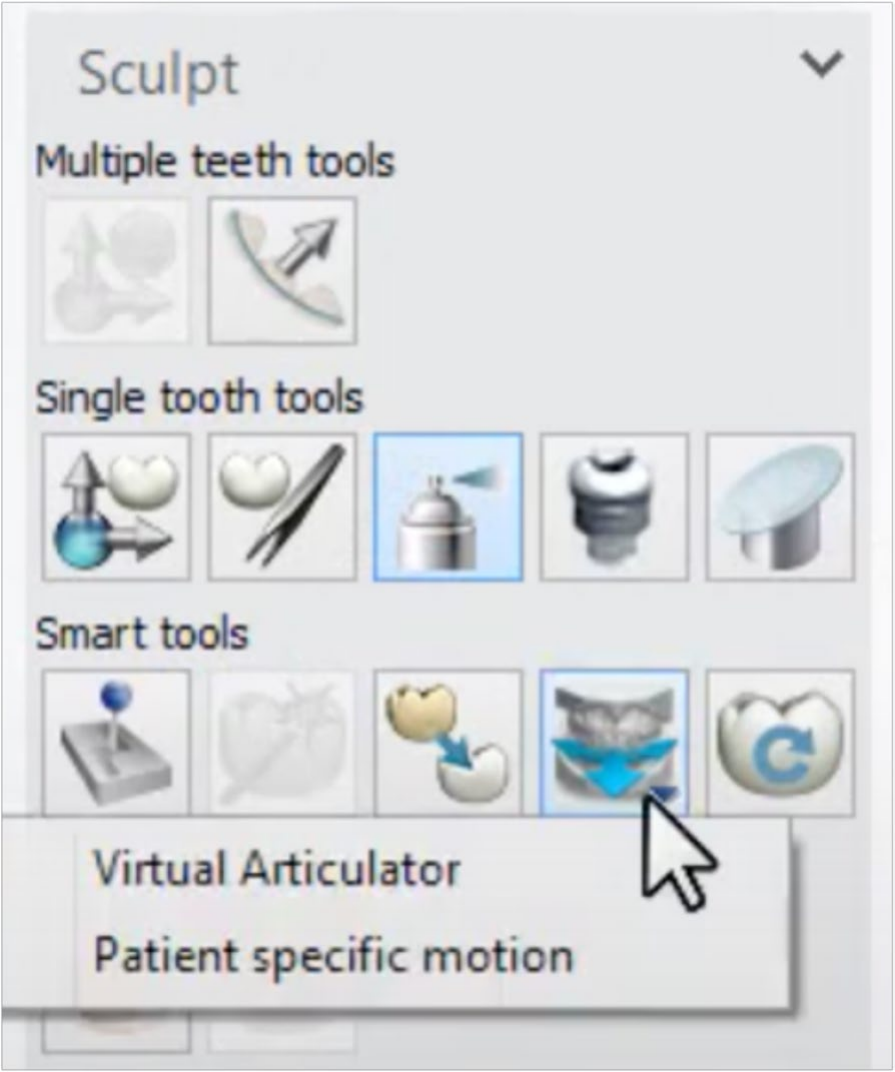
Screenshot from CAD software showing anatomic design for VA and PSM group using “Smart tools”

**Fig. 3:**
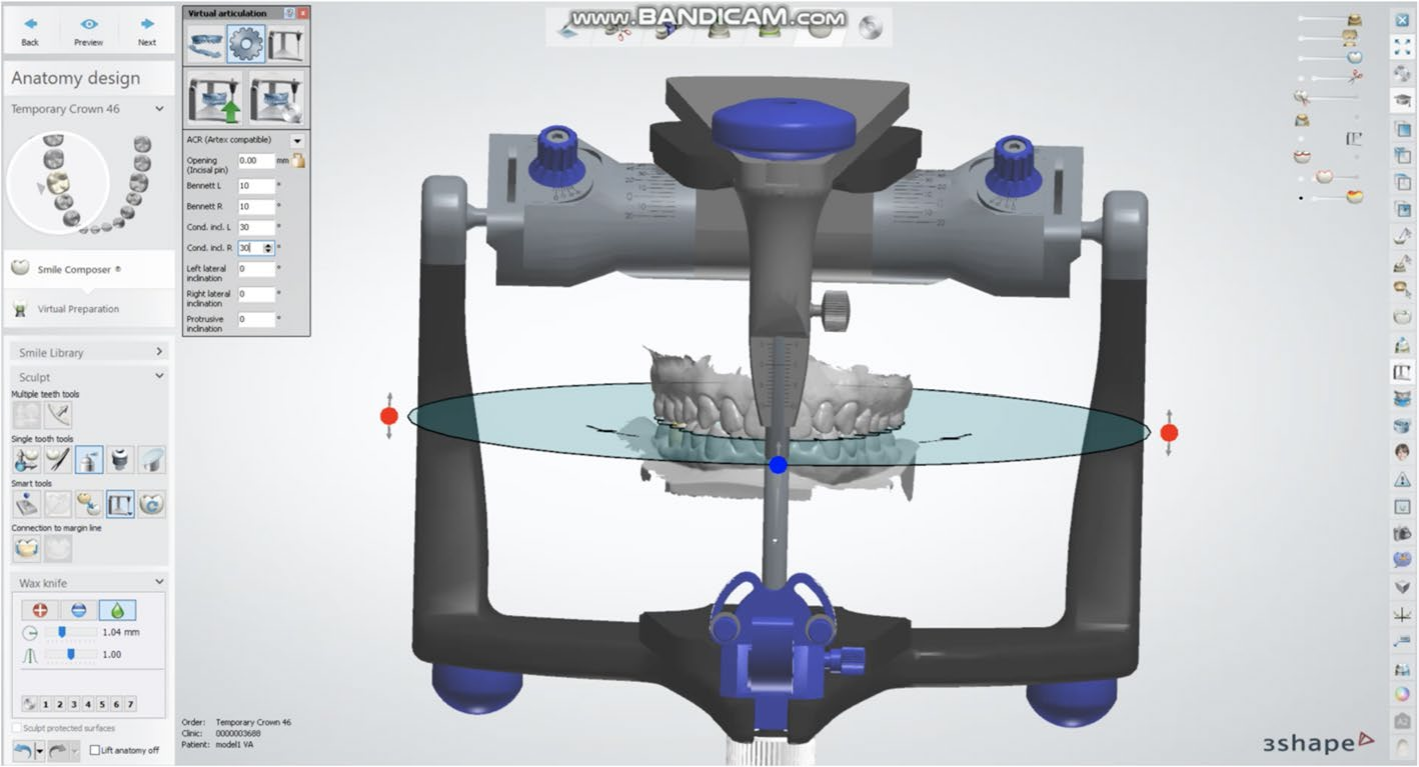
Screenshot of average value setting and occlusal plane registration in CAD software for VA group

**Fig. 4:**
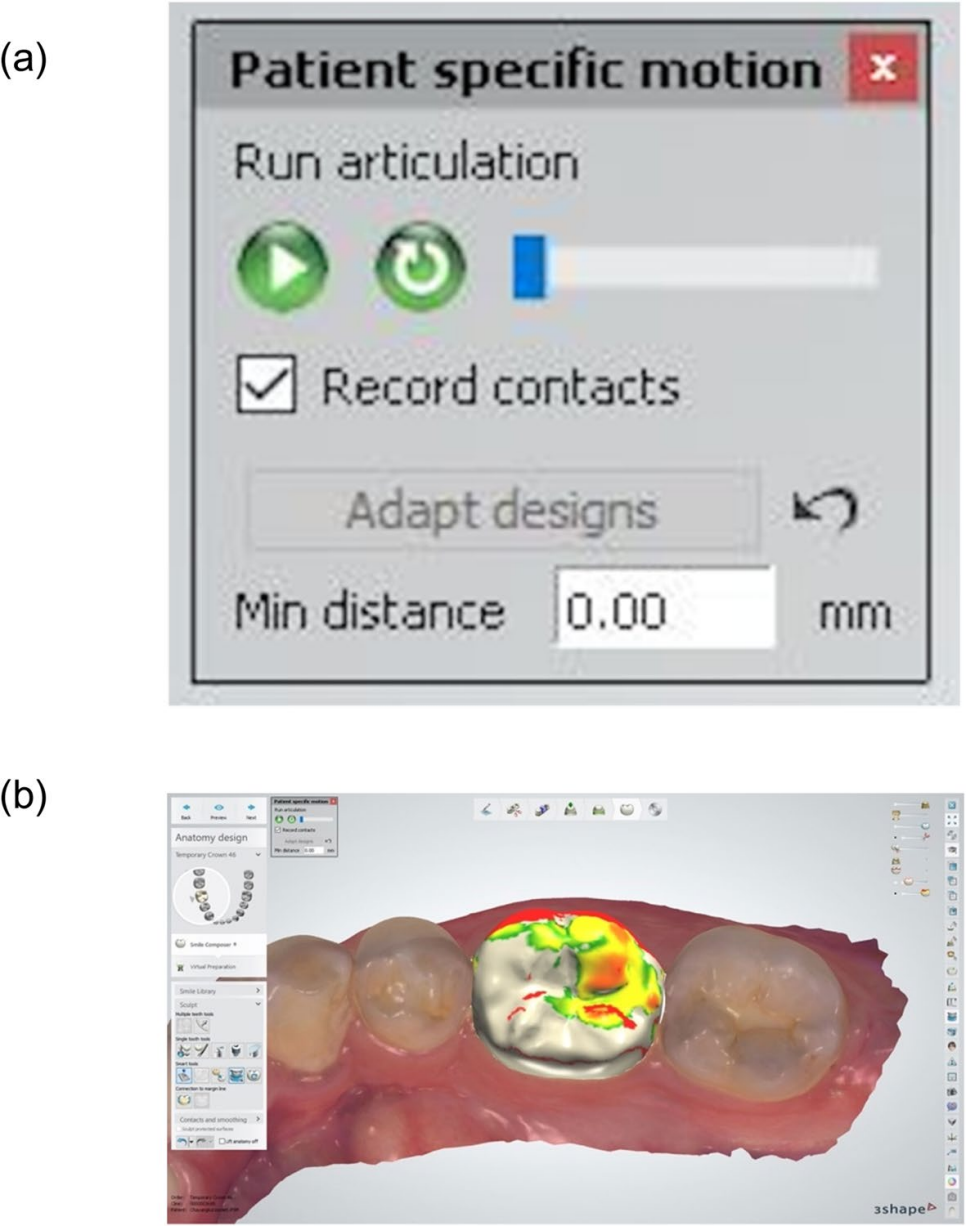
Execution of PSM record on the occlusal surface area. a) PSM feature in CAD software b) Display shown after occlusal registration by PSM

The designed files were standard copied and used to proceed with occlusion registration, with adjustments made using three approaches. Firstly, Static occlusal registration (STA) corrected errors in centric movement. This method used the “Contacts and Smoothing” feature in smart tools to adjust the occlusion statically (Fig. 1). Second, Dynamic occlusal registration was performed using a virtual articulator with average settings (VA) and selecting an Artex CR articulator, configured with mean values: condylar inclination of 30° [9], Bennett angle of 10° [10, 11], IMLT of 0 mm, incisal table inclination of 0°, and incisal pin opening of 0 mm (Figs. 2 and 3). The final method utilized PSM records to capture individualized mandibular movements during eccentric motions, with occlusal registration performed using the virtual motion data obtained during intraoral scanning (Fig. 4).

This process produced three distinct files for each model, representing different occlusal registration methods, resulting in nine experimental files per sample. In total, 90 experimental files were generated for the study (*n* = 10).

#### Obtaining measurements

**Fig. 5:**
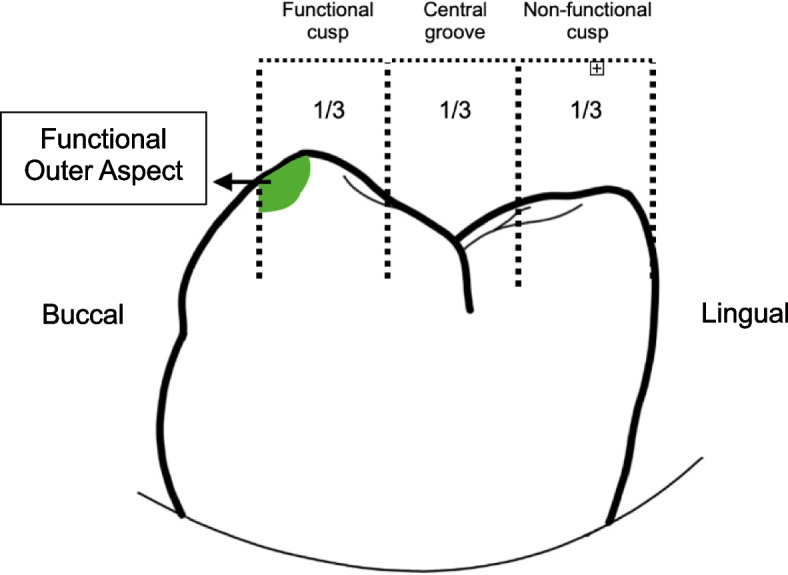
Division of the occlusal surface into three regions (functional cusp, central groove, and non-functional cusp), with highlighted Functional Outer Aspect area located approximately 1 mm from the cusp tip toward the buccal incline

**Fig. 6:**
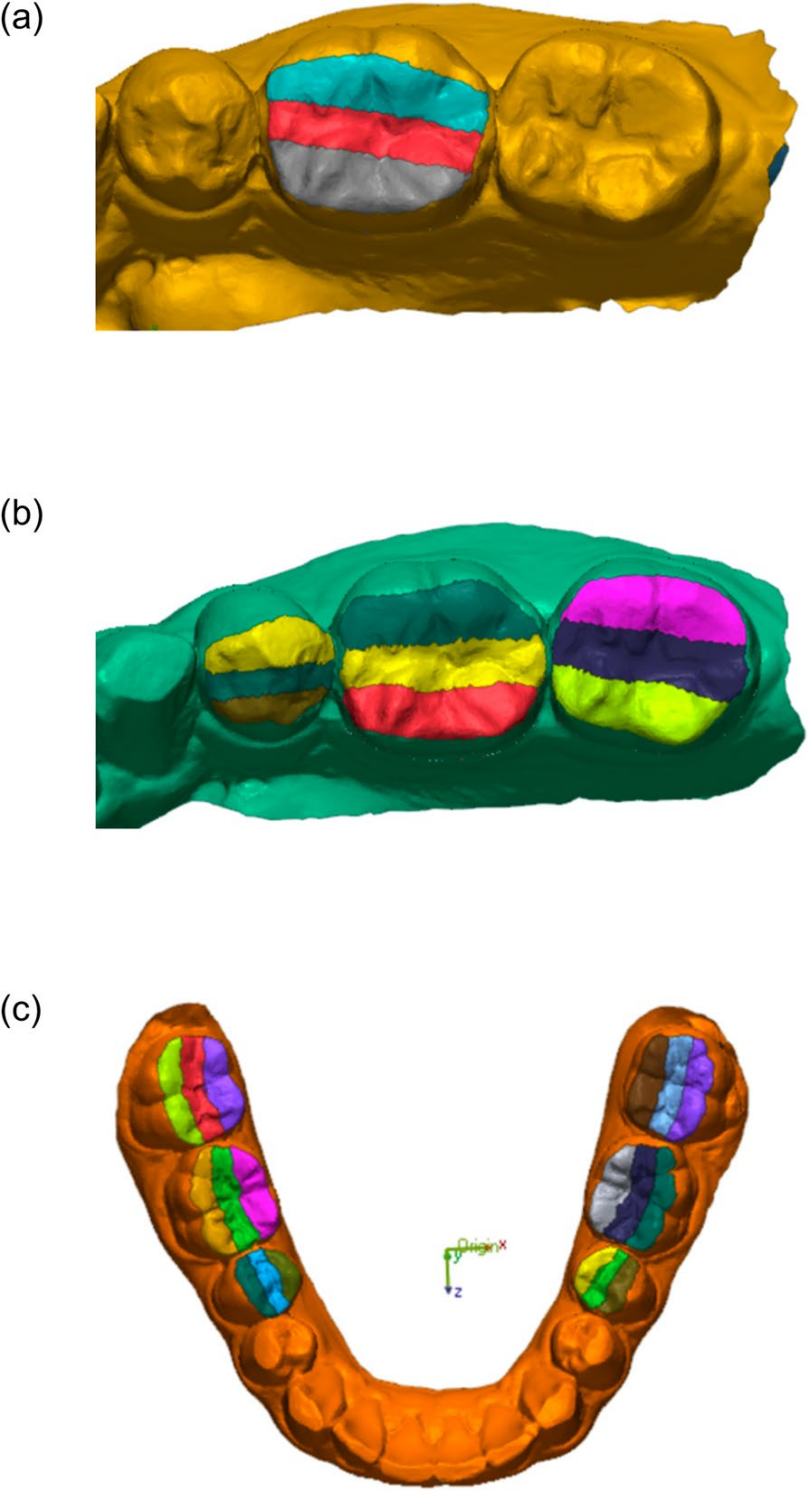
Examples of occlusal surface segmentation in Model 1 (a), Model 2 (b), and Model 3 (c). Each model demonstrates occlusal surface division into three equal regions for analysis

The reference data and experimental groups were imported into a 3-D data-processing software program (Geomagic Control X, version 2018.1.1, 3D Systems Inc., Rock Hill, SC, USA). Deviation measurements of the occlusal surfaces were taken for different occlusal designs, comparing them to the reference data. The steps for 3-D comparison among experimental data were as follows:

1. The original STL scan of each sample was imported as reference data, and the occlusal surface of the posterior teeth was analyzed. The occlusal area was divided into three regions—Functional cusp, Central groove, and Non-functional cusp—by equally dividing the buccolingual width. The functional cusp area included the functional outer aspect of the buccal cusp (approximately 1 mm from the cusp tip toward the buccal incline), the buccal cusp tip, and the lingual incline plane of the buccal cusp. The central groove area covered the occluding region and adjacent inclines, and the non-functional cusp area encompassed the lingual cusp tip and its associated planes (Figs. 5 and 6).

2. The experimental data was aligned with the reference data using the “Alignment Tool”. The “Initial Alignment” established the first correspondence between datasets, followed by “Best-fit Alignment” using a closest-point algorithm to enhance accuracy. The alignment focused on the entire model, excluding the occlusal areas, as the region of interest.

3. The comparison between the reference data and the experimental data was conducted using the"3D Compare Tool”. The comparison focused on specific areas of the occlusal surface, including the Total area, Functional cusp area, Central groove area, and Non-functional Cusp area. Each of these four areas was individually compared against the reference data by selecting the custom region. The specific tolerance value was set to 0.012 mm, and the resulting measurements were reported using the following metrics: Root Mean Square (RMS) value, Standard Deviation (Std Dev), Average (AVG), Average positive deviation (+ AVG), Average negative deviation (-AVG), In Tolerance (In Tol), Out of Tolerance (Out Tol), Over Tolerance (values exceeding the upper tolerance limit), and Under Tolerance (values falling below the lower tolerance limit).

The RMS was calculated by the following Eq. [12]:

$${\varvec{R}}{\varvec{M}}{\varvec{S}}=\sqrt{\frac{{\sum }_{{\varvec{i}}=1}^{{\varvec{N}}}{\left({{\varvec{x}}}_{{\varvec{i}}}\right)}^{2}}{{\varvec{N}}}}$$

where x_i_ is the point distance from the restorations to corresponding point *i* on the original occlusal contacts and *N* is the total number of points.

Subsequently, the experimental data, including STA and dynamic occlusal registration files using VA and PSM, were imported sequentially for the 3D comparison process.

## The resulting measurement data were exported as Excel files for the statistical analysis process.

**Fig. 7:**
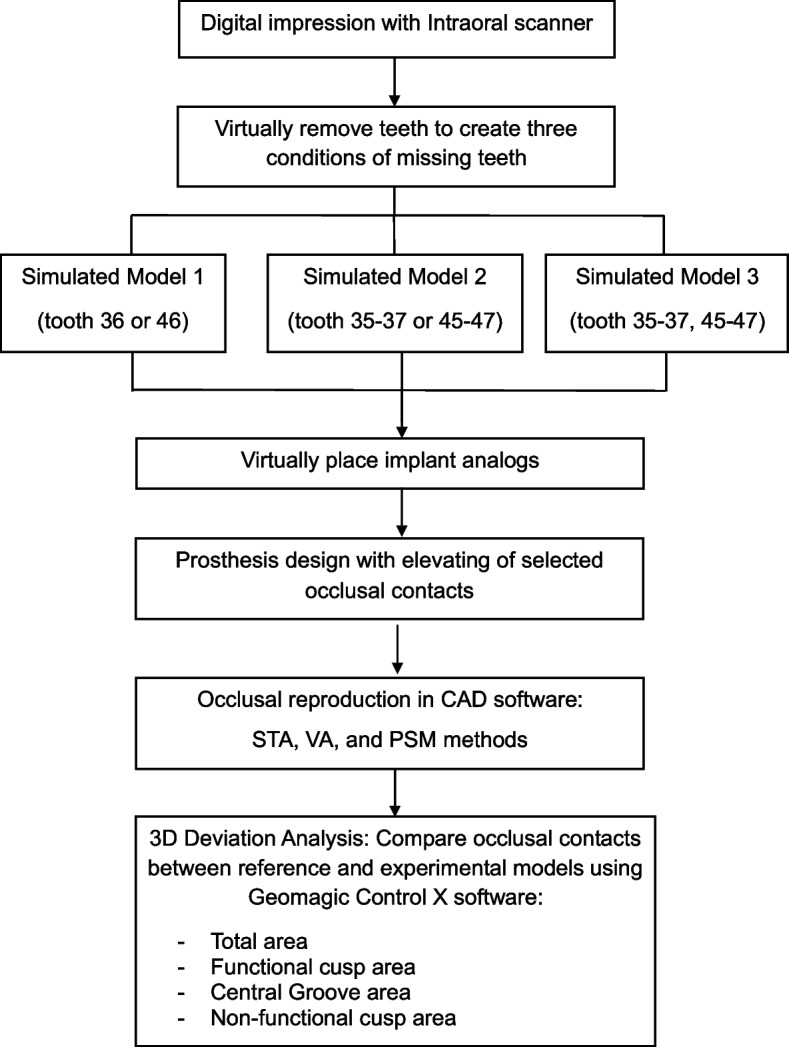
Overview of digital workflow for occlusal reproduction and 3D deviation analysis

An overview of the study workflow, from digital impression acquisition to 3D deviation analysis, is illustrated in Fig. 7. A detailed outline of the digital procedures is provided in Appendix A.

### Data analysis

All statistical analyses were performed using IBM SPSS Statistics Version 28.0. Data normality was assessed using the Shapiro–Wilk test. Three-dimensional variation measurements, including the mean and RMS values, were compared among the three occlusal registration methods (STA, VA, PSM) within each model, and among the three models within each method, using Generalized Estimating Equations(GEE) with pairwise comparisons. Descriptive analysis included the percentage of in-tolerance (In-Tol) and out-of-tolerance (Out-Tol) values, further categorized as over- and under-tolerance, based on a tolerance threshold of ± 0.012 mm. A *p*-value of less than 0.05 was considered statistically significant.

## Result

### Compare static (STA) VS dynamic (VA, PSM) occlusal registration methods

The analysis of RMS values revealed that the PSM group exhibited the highest RMS values across the total occlusal area, as demonstrated in Table 1, suggesting greater variability in measurements compared to other groups. Statistical analyses indicated significant differences in the 3-D deviations of occlusal contacts.

**Table 1:** RMS of three occlusal registration methods (Mean ± SD)(mm.)

Model	Method	Occlusal area			
		**Total**	**Functional cusp**	**Central groove**	**Non-functional cusp**
Model 1	STA	0.164 ± 0.123	0.111 ± 0.076	0.155 ± 0.141	0.168 ± 0.154
	VA	0.167 ± 0.120	0.116 ± 0.071	0.160 ± 0.133	0.170 ± 0.157
	PSM	0.172 ± 0.116	0.121 ± 0.072	0.161 ± 0.139	0.175 ± 0.151
Model 2	STA	0.197 ± 0.111	0.147 ± 0.076	0.217 ± 0.151	0.199 ± 0.118
	VA	0.203 ± 0.108	0.155 ± 0.075	0.222 ± 0.144	0.201 ± 0.120
	PSM	0.206 ± 0.104	0.150 ± 0.074	0.230 ± 0.138	0.205 ± 0.116
Model 3	STA	0.194 ± 0.080	0.170 ± 0.068	0.204 ± 0.112	0.187 ± 0.092
	VA	0.202 ± 0.076	0.185 ± 0.061	0.210 ± 0.110	0.191 ± 0.090
	PSM	0.206 ± 0.074	0.188 ± 0.062	0.219 ± 0.108	0.189 ± 0.087

There was a statistically significant difference between the static (STA) method and both dynamic methods (VA and PSM), with the STA method generally demonstrating lower deviations. Notably, no significant differences were observed between the two dynamic methods (VA vs. PSM) as shown in Table 2.

**Table 2:** Comparison of Mean differences in occlusal registration methods across models

Model	Method	Mean Difference, *p*-Value			
		**Total**	**Functional Cusp**	**Central Groove**	**Non-functional Cusp**
1	STA—VA	−0.004, 0.085	−0.005, 0.190	−0.005, 0.199	−0.002, 0.559
	STA-PSM	−0.009, 0.004*	−0.011, 0.039*	−0.005, 0.327	−0.008, 0.204
	VA—PSM	−0.005, 0.059	−0.006, 0.071	0.000, 0.986	−0.006, 0.300
2	STA—VA	−0.006, 0.014*	−0.008, 0.116	−0.005, 0.165	−0.003, 0.212
	STA-PSM	−0.008, 0.064	−0.003, 0.514	−0.013, 0.088	−0.006, 0.049*
	VA—PSM	−0.003, 0.519	0.005, 0.420	−0.008, 0.240	−0.004, 0.214
3	STA—VA	−0.008, < 0.001*	−0.015, 0.001*	−0.006, 0.001*	−0.004, 0.022*
	STA-PSM	−0.012, 0.015*	−0.018, 0.001*	−0.016, 0.019*	−0.002, 0.569
	VA—PSM	−0.004, 0.354	−0.003, 0.425	−0.010, 0.102	0.002, 0.641

^*^Represent statistical significant difference (*p* < 0.05)

In Model 1, significant differences were observed between STA and PSM in the total area and functional cusp, with *p*-values of 0.004 and 0.039, respectively. Model 2 highlighted significant differences between STA and VA in the total area with a *p*-value of 0.014 and between STA and PSM in the non-functional cusp area with a *p*-value of 0.049. Model 3 showed comprehensive differences, where STA differed significantly from VA across all measured areas: Total (*p* < 0.001), Functional Cusp (*p* = 0.001), Central Groove (*p* = 0.001), and Non-functional Cusp (*p* = 0.022). Significant differences were also noted between STA and PSM in the Total area (*p* = 0.015), Functional Cusp (*p* = 0.001), and Central Groove (*p* = 0.019).

The In-Tolerance (In-Tol) and Out-of-Tolerance values included over and under tolerance values of different occlusal registration methods (In-tolerance value setting as 0.012 mm).

### The In-Tolerance (In-Tol) and Out-of-Tolerance values included over and under tolerance values of different occlusal registration methods (In-tolerance value setting as 0.012 mm)

The percentage of surface points falling within the acceptable tolerance range (In-Tolerance) was evaluated, along with those exceeding the upper (Over-Tol) and lower (Under-Tol) tolerance limits. The results presented in the following tables provide a comparative overview of the methods within each model. The data are expressed as percentages, emphasizing the proportion of surface points in each category. From Table 3, it is observed that in Model 1, the PSM method exhibits a higher percentage of over-tolerance in the functional cusp, central groove, and non-functional cusp areas compared to the other methods. In contrast, Model 2, shows that the PSM method has a higher percentage of under-tolerance in the functional cusp and central groove areas while the VA method shows a higher percentage of over-tolerance in those areas. Additionally, Model 3, indicates that the PSM method demonstrates a higher percentage of under-tolerance across all occlusal areas while the VA method shows a higher percentage of over-tolerance in the same area.

**Table 3:** Mean percentage of In-tolerance, Out-of-tolerance over, and Out-of-tolerance under of Model 1, Model 2, and Model 3

Model	Area	Methods	In-Tol	Over-Tol	Under-Tol
Model 1	Total	STA	44.53	16.96	38.50
		VA	38.58	20.07	41.35
		PSM	44.64	18.56	36.80
	Functional Cusp	STA	35.95	18.74	45.31
		VA	37.62	20.32	42.07
		PSM	36.01	20.97	43.01
	Central Groove	STA	30.34	26.52	43.14
		VA	30.36	28.32	41.32
		PSM	30.07	28.80	41.12
	Non-functional Cusp	STA	48.81	12.38	38.81
		VA	47.29	12.42	40.28
		PSM	48.14	14.90	36.96
Model 2	Total	STA	25.18	29.21	45.61
		VA	25.19	29.07	45.74
		PSM	25.32	27.87	46.80
	Functional Cusp	STA	23.35	28.07	48.57
		VA	25.97	28.09	45.93
		PSM	25.03	25.14	49.82
	Central Groove	STA	20.16	33.76	46.07
		VA	20.07	35.31	44.62
		PSM	18.79	34.12	47.08
	Non-functional Cusp	STA	31.99	25.60	42.41
		VA	29.71	23.56	46.73
		PSM	32.22	23.93	43.85
Model 3	Total	STA	28.90	27.68	43.42
		VA	27.99	28.67	43.34
		PSM	27.00	27.31	45.69
	Functional Cusp	STA	27.58	24.63	47.78
		VA	26.41	25.88	47.72
		PSM	25.47	25.24	49.29
	Central Groove	STA	23.13	35.64	41.22
		VA	22.34	36.84	40.81
		PSM	20.91	34.88	44.21
	Non-functional Cusp	STA	35.74	22.60	41.67
		VA	35.04	23.03	41.93
		PSM	34.47	21.31	44.21

### The effect of the number of missing teeth within each method

Additional analysis comparing different models of missing teeth (Model 1 vs. Model 2, Model 1 vs. Model 3, and Model 2 vs. Model 3) within each occlusal reproduction method revealed that comparisons between Model 1 and Model 3, as well as between Model 2 and Model 3, demonstrated statistically significant deviations (*p* < 0.05). Significant differences were primarily observed at the functional cusp areas across all occlusal reproduction methods (SA, VA, and PSM). Additionally, in the central groove area, a significant difference was found for the PSM method between Model 1 and Model 2 (*p* = 0.042). Notably, the mean differences indicated a trend of increasing deviation magnitude with the extent of missing teeth, following the pattern Model 3 > Model 2 > Model 1. Pairwise comparisons of occlusal deviations among different models are summarized in Table 4.

**Table 4:** Pairwise comparisons of RMS value in different models of missing teeth across occlusal registration methods and occlusal areas

Area	Method	*p*-Value		
		**Model1 VS Model 2**	**Model1 VS Model3**	**Model2 VS Model3**
Total	SA	0.204	0.186	0.845
	VA	0.161	0.124	0.955
	PSM	0.181	0.149	0.974
Functional cusp	SA	0.216	0.036*, -.059350^a^	0.036*, -.022980^a^
	VA	0.202	0.017*, -.069160^a^	0.006*, -.029960^a^
	PSM	0.350	0.025*, -.066430^a^	0.001*, -.037870^a^
Central groove	SA	0.090	0.089	0.546
	VA	0.084	0.086	0.569
	PSM	0.042*, -.069370^a^	0.051	0.627
Non-functional cusp	SA	0.328	0.514	0.514
	VA	0.319	0.487	0.600
	PSM	0.330	0.633	0.415

^*^Represent statistical significant difference (*p* < 0.05)

^a^Represent mean difference

### The color-code assessment in the occlusal area of all model

**Fig. 8:**
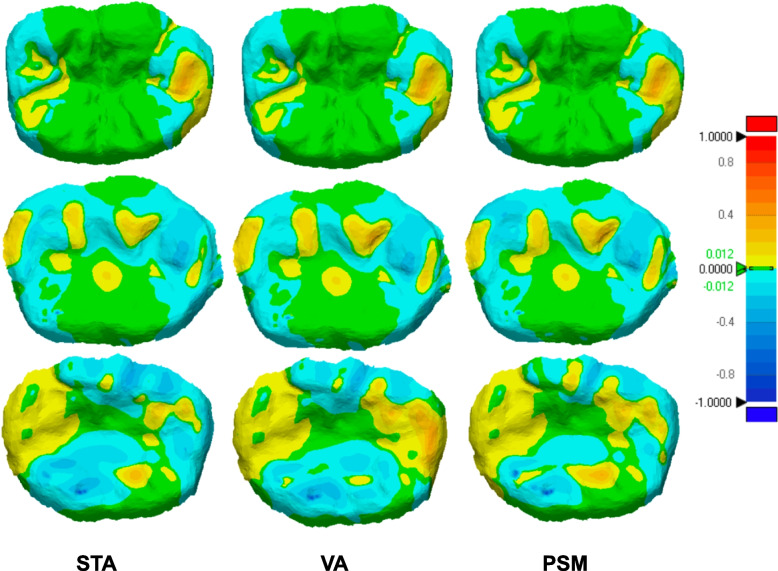
Visual representations of color-coded maps showing deviation among STA, VA, and PSM in Model 1. The tolerance value was set at 0.012 mm for deviation analysis

**Fig. 9:**
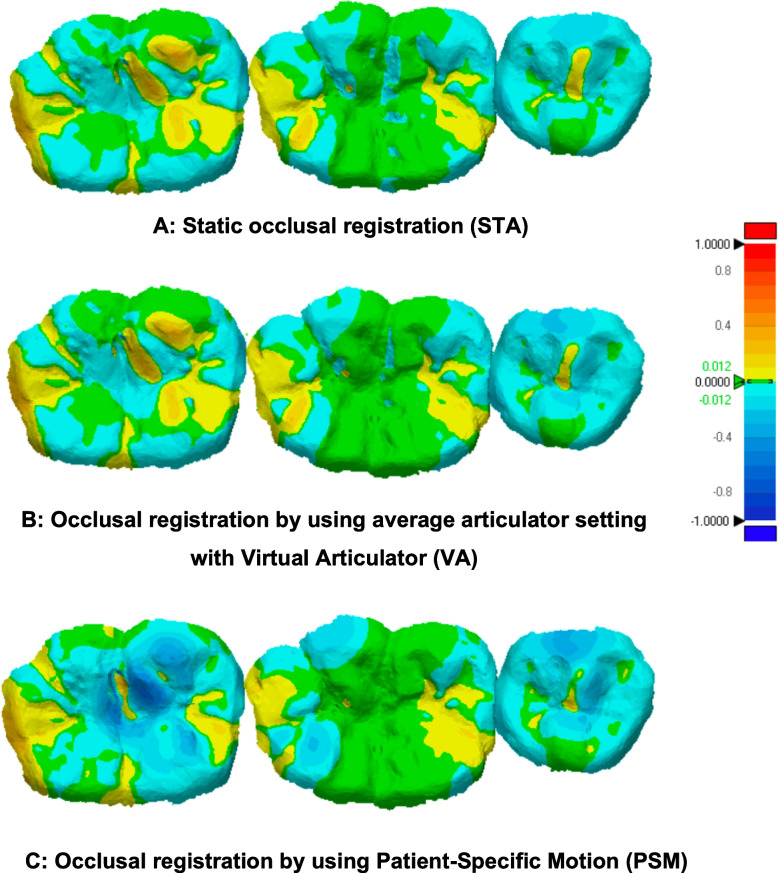
Color-coded maps showing deviation among (A) STA, (B) VA, and (C) PSM in Model 2. The tolerance value was set at 0.012 mm

**Fig. 10:**
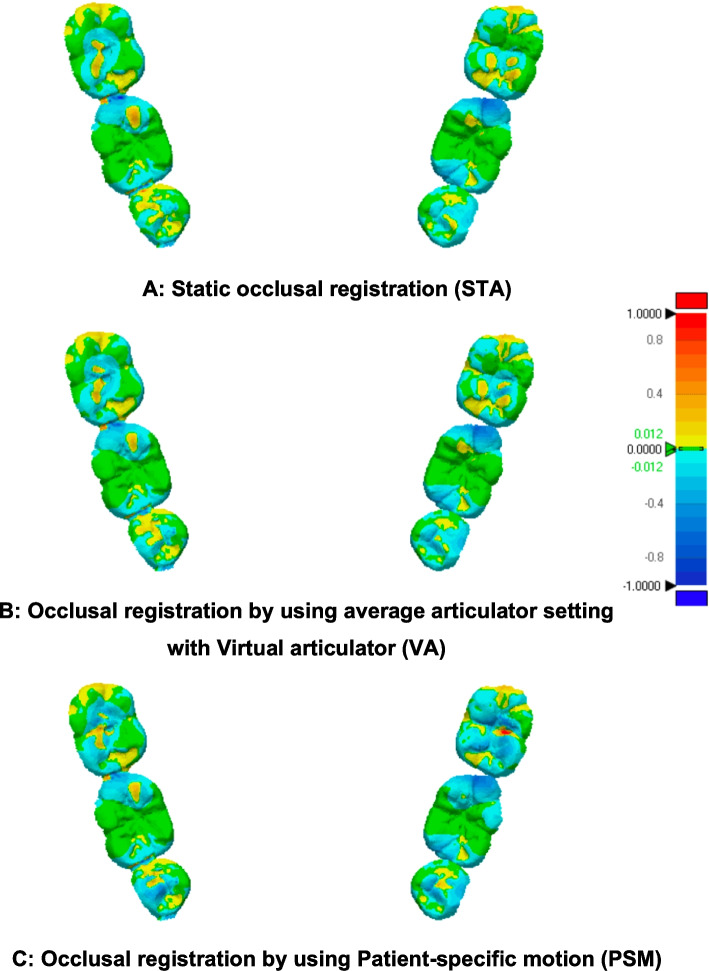
Color-coded maps showing deviation among (A) STA, (B) VA, and (C) PSM in Model 3, The tolerance value was set at 0.012 mm

The qualitative analysis results (Figs. 8, 9 and 10) revealed that most of the occlusal surface in all three groups was coded green, indicating minimal deviation and that the majority of the occlusal surface is within acceptable limits. The central groove, serving as the main contact area during centric occlusion, was predominantly green and yellow. The yellow and blue coded areas, representing occlusal errors, were primarily located on cusp tips and inclined planes. In most cases, the PSM group showed a negative deviation compared to the STA and VA groups (Figs. 9, and 10), with some cases showing extensive negative deviation areas indicative of overcorrection (Fig. 10C). In contrast, the VA group showed an increase in the yellow-coded area compared to the STA and PSM group especially in functional cusp and central groove areas especially in model 2 and 3.

Overall, PSM showed a trend of negative deviations, while the VA showed the largest positive deviations among all occlusal registration methods.

**Fig. 11:**
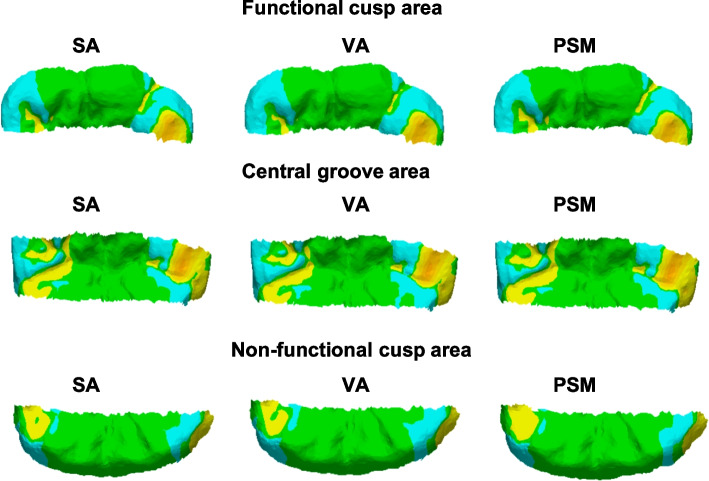
Visual representations of color-coded maps showing deviation of Model1 in different occlusal areas

For quality assessment, an example of the divided surface is shown in Fig. 11.

## Discussion

Proper occlusion is essential in implant-supported restorations to prevent mechanical and biological complications [13]. This study critically evaluated the efficacy of various occlusal registration methods within a digital workflow, aiming to enhance prosthesis design by accurately reflecting dynamic occlusal relationships.

In this study, the occlusal surfaces were designed using three methods: STA, VA, and PSM. The 3-D deviation between the designed occlusal surfaces and the original occlusal surface was subsequently analyzed. The null hypothesis was rejected, as significant differences were observed among the different occlusal registration methods and the conditions of the missing teeth. The findings revealed a significant difference between the static and dynamic methods (STA vs. VA and STA vs. PSM). Crowns designed using the PSM method showed the highest 3D deviation values. However, no significant difference was found between the two dynamic methods, VA and PSM.

The STA crowns in this study were primarily designed based on static occlusion, which does not account for mandibular movements and may lead to occlusal interferences during functional excursions. These crowns were automatically adjusted using the built-in algorithm of the dental CAD software, and this group exhibited the lowest RMS values, suggesting minimal deviation from the reference. In contrast, the VA method incorporates virtual simulation of jaw movements by aligning digital maxillary and mandibular models, initially positioned by software and then refined according to an average occlusal plane. Although VA is designed to enhance functional occlusal accuracy, it relied on average-value articulator settings in this study and did not capture patient-specific mandibular movements. The effectiveness of VA is therefore highly dependent on the precision of the intraoral scans and the assumptions of the software algorithm. In, this study, VA exhibited greater positive deviations than STA, indicating a tendency toward under-corrected occlusal contacts, which can lead to an increase in the clinical chair time required for adjustments. The unexpected finding that VA produced larger positive deviations than STA could be attributed to the fact that STA design based on patient’s actual bite force during scanning, potentially resulting in tighter occlusal contacts. Meanwhile, VA’s automated adjustments, using average articulator settings and undisclosed algorithmic parameters, may fail to replicate individual functional dynamics. These findings highlight the limitations of software-based articulation in accurately reproducing patient-specific occlusal morphology.

With the development of CAD/CAM technology, digital workflows have incorporated PSM, which involves recording a patient’s individualized jaw movements to create a customized prosthesis. The detailed data captured by the intraoral scanner is crucial for accurately replicating these movements. Nevertheless, the manufacturer has not revealed the fundamental principles behind the PSM function, and limited research exists to determine if digital workflows utilizing digitally simulated dynamic occlusion are clinically more effective than traditional methods [14]. In addition, compared to conventional jaw motion tracking systems, such as the Zebris JMA-Optic, Modjaw 4D Motion Capture System, and Kavo Arcus Digmall [15], the PSM function offers a simplified, fully digital alternative by integrating dynamic motion capture directly into the intraoral scanning and CAD workflow. While traditional systems may provide more comprehensive movement data, they are often technique-sensitive and require additional hardware. In contrast, the PSM approach improves clinical efficiency by eliminating the need for external tracking devices, though it may have limitations in accuracy in certain conditions, such as in patients with compromised occlusal support or extensive edentulism.

In PSM recording, the alignment of maxillary and mandibular casts typically relies on buccal surfaces of a few teeth or selected reference points, which may introduce alignment errors [8]. Additionally, digital casts are scanned without occlusal force, while the buccal occlusion data in maximum intercuspal position is scanned under loading. This mismatch may lead to discrepancies in tooth position due to bone distortion [16, 17] and physiological tooth displacement during occlusal loading [18]. Because digital models cannot accurately simulate these functional changes, the software may overestimate contact areas. This can result in restorations with fewer or no actual occlusal contacts, increasing the risk of supraocclusion of the opposing teeth [8].

RMS was used as an indicator of the extent to which deviations between two datasets differ from zero. In this study, after accounting for and eliminating all systematic errors, the only remaining deviations between the reference data and the comparison datasets (STA, VA, and PSM) were attributed to the sum of points where occlusal adjustment were necessary. Consequently, the RMS value reflected the average magnitude of occlusal error in the comparison area. Although the PSM method exhibited the highest deviation as indicated by the RMS values, the mean difference was only about 6–18 µm, which is minimal and clinically insignificant. Furthermore, research literature that examines and supports 3-D deviation measurement suggests that RMS does not indicate clinical significance [19]. It simply reflects the degree of difference without revealing the trend or direction (e.g., improvement or worsening, increase or decrease).

While this study was conducted in the context of implant-supported restorations, the techniques evaluated and principles of occlusal registration are also applicable to tooth-supported prostheses. Accurate occlusion is critical across all types of restorations to ensure proper function, reduce complications, and enhance patient comfort. Therefore, the findings may offer clinical guidance beyond implant dentistry. To apply these findings in a clinical setting, dental professionals typically use specific tools such as articulating paper (ranging in thickness from 40 to 200 µm) to check occlusion, as well as shimstock (usually around 8–12 µm thick) [20] to assess the tightness of the occlusion. In the design of occlusal surfaces for crowns and bridges on natural teeth, it is often desirable to achieve a tight occlusion with no interferences. However, for implant-supported prostheses, centric occlusion should exhibit light contact sufficient for the shimstock to pass with slight resistance. Additionally, there should be no interferences during eccentric movements [13]. In this study, a tolerance value of 0.012 mm, corresponding to the thickness of shimstock, was set to reflect clinically relevant conditions. The results indicate that the PSM method tends to produce over-tolerance in Model 1, leading to slightly elevated occlusal surfaces. As the complexity increases, particularly in Model 2, the PSM method shifts towards under-tolerance, especially in the functional cusp and central groove areas. In the most complex scenario (Model 3), the PSM method consistently underperforms, showing a higher percentage of under-tolerance across all areas. Meanwhile, the VA method tends to show over-tolerance, leading to higher restorations. Given these findings, the PSM method may be more suitable for cases with less tooth loss, where maintaining precise occlusal contact is critical. However, in more complex cases, where tooth loss is significant, the PSM method might require closer monitoring to avoid under-occlusal reproduction that could lead to insufficient occlusal contact. In addition, the findings indicate that as the number of missing teeth increases, the challenge of accurately reproducing occlusal morphology also increases, particularly at the functional cusp areas. The observed trend, with greater deviations in Model 3 compared to Model 2 and Model 1, underscores the sensitivity of occlusal reproduction to the extent of posterior support loss. Significant deviations at the functional cusp and central groove areas further highlight the critical role of remaining dentition in maintaining occlusal stability.

Color-coded assessment was used in this study to visualize the magnitude and distribution of positive and negative deviations between the experimental and reference data. This approach clearly illustrates occlusal discrepancies at clinically relevant contact areas. Typically, the central groove is involved in centric occlusion, the functional cusp area in mediotrusive movement, and both functional and non-functional cusps during laterotrusive movement [21]. In all three methods (STA, VA, and PSM), comparable patterns were observed. Yellow-coded regions, commonly seen on cusp tips, indicate slight elevations relative to the reference—an expected finding, as cusp tips are primary contact points. This observation aligns with Lee et al., 2020 [6], which also found that occlusal errors were most prevalent on cusp tips and inclined planes. Notably, the PSM group showed more blue-coded (negative deviation) areas on both functional and non-functional cusps, suggesting slight under-occlusion. This may be advantageous in implant-supported prostheses by minimizing the risk of eccentric contacts and reducing direct occlusal force on implants, contributing to long-term stability.

These findings are consistent with previous studies. Lee et al., 2020 [6] and Li et al., 2023 [8] reported that the PSM method yielded the least occlusal deviation, likely due to its ability to replicate patient-specific mandibular movements and wear facet morphology accurately. However, both studies focused primarily on single-unit crowns. In contrast, the present study investigated more complex prosthetic scenarios using long-span fixed partial dentures across varying conditions of missing teeth. While PSM remained generally effective, its accuracy declined as occlusal complexity increased, particularly in cases with extensive tooth loss. This may reflect the reduced ability of PSM to simulate functional occlusion when occlusal support is compromised. Moreover, the lower accuracy of intraoral scanners in edentulous areas, due to the absence of distinct anatomical landmarks needed for precise image stitching and alignment, may further compromise the reliability of PSM recordings. Therefore, despite its advantages, the use of PSM in cases with limited occlusal support should be approached with caution, and additional verification methods may be necessary to ensure clinical reliability.

This study employed a fully digital workflow to simulate clinical conditions where occlusal registration plays a critical role and individual patient variability must be considered. Fully dentate participants were selected to ensure stable reference points and enhance the accuracy of deviation analysis. While this approach allowed for controlled comparisons, it also introduced certain several limitations. First, the PSM recording process is confined to a closed system, requiring the TRIOS intraoral scanner, 3Shape Dental System CAD software, and outputs data in the proprietary 3oxz format. This restricts workflow flexibility and compatibility with other platforms. However, the overall methodology can be adapted to alternative CAD/CAM systems that support dynamic occlusion and 3D deviation analysis. Alternative intraoral scanners, such as the Medit i700 (Medit Corp.), Primescan (Dentsply Sirona) integrated with SICAT Function software, or TRIOS 4 (3Shape A/S), could be employed following similar principles. Likewise, CAD platforms such as exocad DentalCAD (exocad GmbH), Dental Wings Open Software (Dental Wings Inc.), or Sirona inLab (Dentsply Sirona) offer virtual articulation features suitable for dynamic occlusion design. If PSM recording is not available, alternative jaw motion tracking devices, including SICAT Function and Modjaw, may be incorporated to capture individualized mandibular movements. The selection of software in this study was based on its widespread clinical relevance, precision, and availability. To further enhance reproducibility, a detailed outline of the digital procedures has been provided in Appendix A. Additionally, since this study was conducted entirely within a digital environment, the results may not fully reflect real clinical scenarios. Factors such as laboratory processing, material properties, and individual patient’s conditions can affect prosthesis accuracy. Deviations observed in this protocol represent only part of the potential cumulative errors in clinical workflows. Future in-vivo studies are recommended to validate these findings, particularly with respect to occlusal adjustment time, patient satisfaction, cost-effectiveness, and prosthesis designs, including anterior or maxillary posterior restorations, as well as the influence of various occlusal schemes on functional outcomes. Finally, although statistical consultation confirmed that a sample size of ten participants was sufficient to achieve the objectives of this study, future studies with larger sample sizes are recommended to improve the generalizability of the findings and support broader clinical applications.

## Conclusion

1. This study confirms the significant impact of various occlusal registration methods like STA, VA, and PSM on prosthesis design, particularly customized to the patient’s existing dental conditions.

2. Despite differences among these methods, all outcomes fall within clinically acceptable ranges, supporting their practical application in prosthodontic workflows to reduce the need for clinical occlusal adjustments.

3. The findings highlight a careful selection of occlusal registration methods based on individual patient needs, particularly highlighting PSM for its ability to customize prosthesis design and potentially enhance treatment outcomes.

4. A reduction in remaining dentition led to increased occlusal deviation across all occlusal registration methods, particularly at the functional cusp areas. These findings underscore the importance of preserving occlusal support to maintain accuracy in digital prosthesis design, especially in partially edentulous cases.

## Supplementary Information

Additional file 1.

## Data Availability

The datasets used and/or analysed during the current study are available from the corresponding author on reasonable request.

## Abbreviations

CAD/CAM: Computer-aided design and computer-aided manufacturing
STA: Static occlusal registration
STL: Standard tessellation language
VA: Average setting of Virtual articulator
PSM: Patient Specific Motion
MA: Mechanical Articulator
