# Supplementary material for: Accuracy of individual mandibular motion records using intraoral scanner for fixed implant- supported prosthesis designs: a comparative study
Source: BMC Oral Health. 2025 Jun 2;25:878. doi: 10.1186/s12903-025-06282-x (PMC12128537; doi:10.1186/s12903-025-06282-x)
Supplement: Supplementary file 1 — Additional file 1. [file 12903_2025_6282_MOESM1_ESM.docx]

**Appendix A**. Step-by-Step Digital Workflow Setup for 3D deviation analysis

1. Intraoral Scanning (TRIOS 3 Intraoral Scanner; 3Shape A/S, Copenhagen, Denmark)

- Launch the 3Shape Dental System software and select “New Order”
- Choose “Scan Only” mode
- Perform the intraoral scan:
- Scan the maxillary arch
- Scan the mandibular arch
- Perform a buccal bite scan (with the patient in maximum intercuspation both left and right sides)
- Select the "Patient Specific Motion" recording mode and capture: Right and left lateral movements, Protrusive movement, with scanner wand positioned on the buccal surfaces of the first molars on the group function side
- Export STL files for both arches and dynamic motion data (.3oxz format) via the Order Manager

2. Manipulation of Digital Dentition (Model Builder and Implant Studio modules of 3 shape Dental System, version 2019; 3Shape A/S, Copenhagen, Denmark)

- Import the scanned .3OXZ files
- Virtually remove teeth and place implant analogs to create three different models:
- Model 1: First molar (unilateral)
- Model 2: Second premolar, first molars, and second molar (unilateral)
- Model 3: Second premolar, first molar, and second molar (bilateral)

3. Prosthesis Design (3Shape Dental System v2019; 3Shape A/S, Copenhagen, Denmark)

- Import the prepared model files and categorize cases as implant-supported crowns
- Adjust occlusal morphology and crown contours to align anatomically with adjacent and opposing teeth
- Visualize occlusal contacts using the color-coded contact map: identify the tightest contacts (orange to red zones)
- Elevate selected occlusal contacts points using the Sculpting Tool (set at 0.3 mm intensity)
- Save each crown design individually

4. Occlusal Reproduction

- Copy the designed crown files and reprocess each one according to the assigned registration method
- STA: use "Contacts and Smoothing" feature under Smart Tools for static occlusal registration
- VA: Activate the "Virtual Articulator" with Artex CR articulator, configured with mean values: Condylar inclination of 30°, Bennett angle of 10°, IMLT of 0 mm, incisal table inclination of 0°, and incisal pin opening of 0 mm
- PSM: Activate the “Patient Specific Motion”
- Export the resulting occlusal reproduction files as STL files

5. 3D Deviation Analysis (Geomagic Control X, version 2018.1.1, 3D Systems Inc., Rock Hill, SC, USA)

- Open Geomagic Control X software
- Import the original reference scan and crown designs for each experimental group
- Segment the occlusal surface into three regions of interest: Functional cusp, Central groove, and Non-functional cusp areas.
- Perform 3D alignment:
- Use "Initial Alignment" followed by "Best-fit Alignment". The alignment was focused on the entire model, excluding the occlusal surfaces to isolate deviations
- Compare the occlusal surfaces using the “3D Compare” tool:
- Evaluate each occlusal region against the reference scan
- Set tolerance limits at ±0.012 mm.
- Generate color-coded deviation maps
- Extract Root Mean Square (RMS) values and export deviation reports
